# Supplementary material for: The Role of Fractalkine in the Regulation of Endometrial Iron Metabolism in Iron Deficiency
Source: Int J Mol Sci. 2023 Jun 8;24(12):9917. doi: 10.3390/ijms24129917 (PMC10298019; doi:10.3390/ijms24129917)
Supplement: Supplementary file 1 [file ijms-24-09917-s001.zip › ijms-2425606-supplementary.pdf]

The Role of Fractalkine in the Regulation of Endometrial Iron Metabolism at Iron Deficiency

Edina Pandur [1,2,*], Ramóna Pap [1,2], Gergely Jánosa [1], Adrienn Horváth [1] and Katalin Sipos [1,2]

[1] Department of Pharmaceutical Biology, Faculty of Pharmacy, University of Pécs, H-7624 Pécs, Hungary; pap.ramona@pte.hu (R.P.); janosa.gergely@gytk.pte.hu (G.J.); adrienn.horvath2@gytk.pte.hu (A.H.); katalin.sipos@aok.pte.hu (K.S.)
[2] National Laboratory on Human Reproduction; University of Pécs, H-7624 Pécs, Hungary
* Correspondence: edina.pandur@aok.pte.hu

Figure 3A

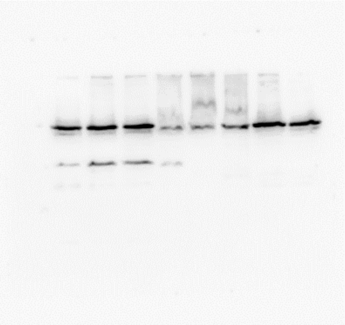

DMT-1

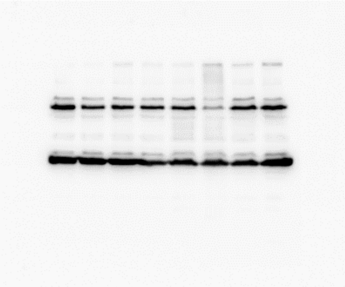

FP (up), GAPDH (down)

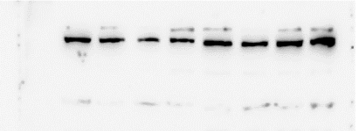

TfR1 (up)

Figure 4A

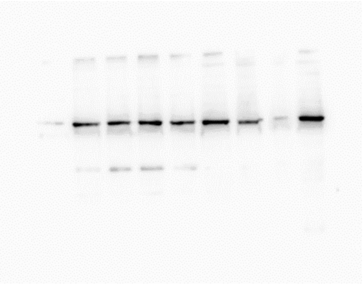

DMT-1

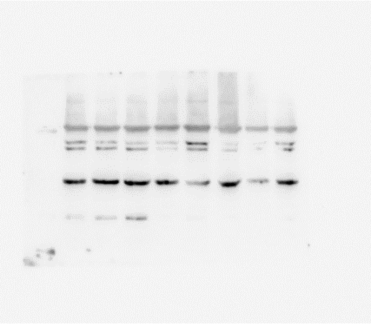

FP (down)

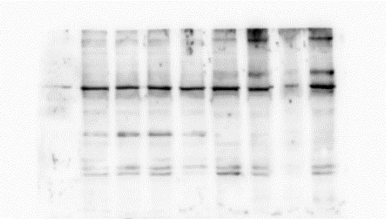

TfR1

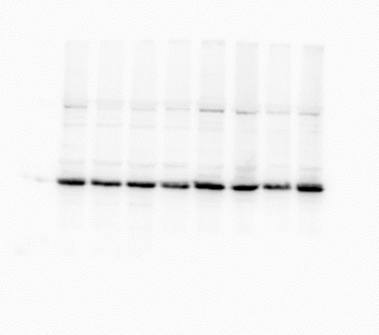

GAPDH

Figure 6A

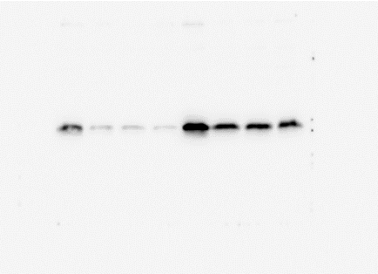

FTH

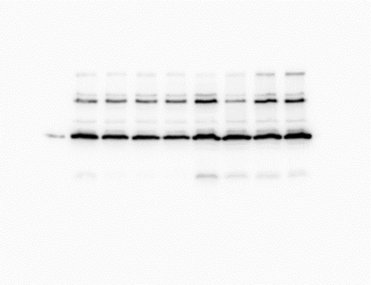

GAPDH

Figure 6C

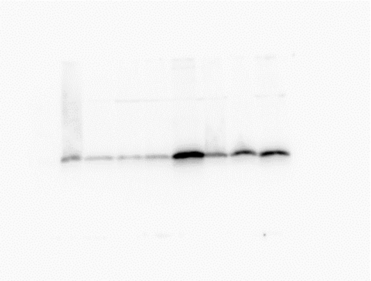

FTH

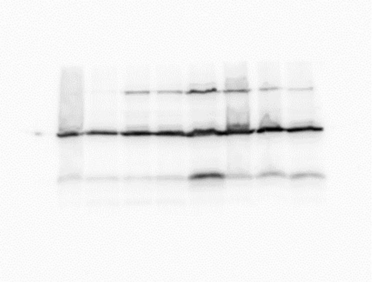

GAPDH

Figure 8A

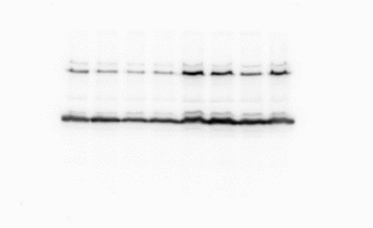

FECH (up), GAPDH (down)

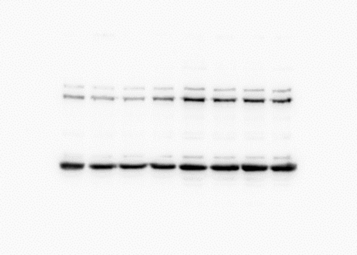

GAPDH (down)

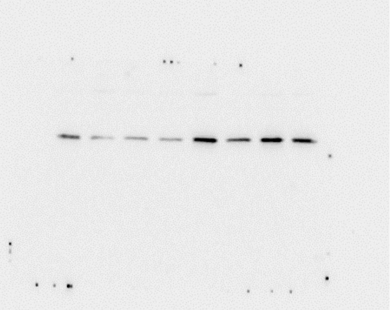

HO-1

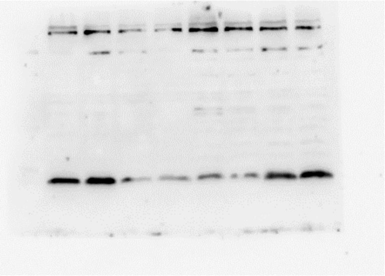

Mfrn-1

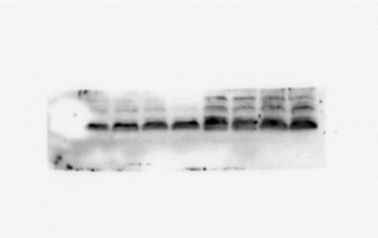

Mfrn-2

Figure 9A

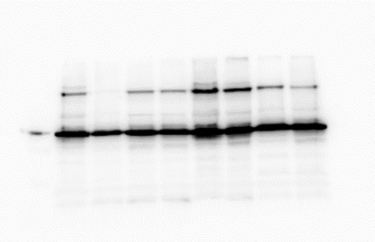

FECH (up), GAPDH (down)

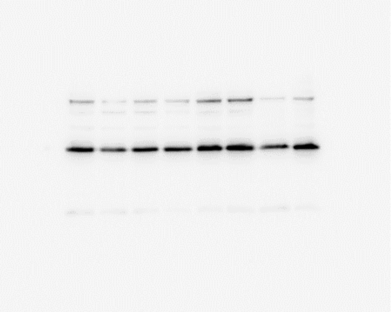

GAPDH

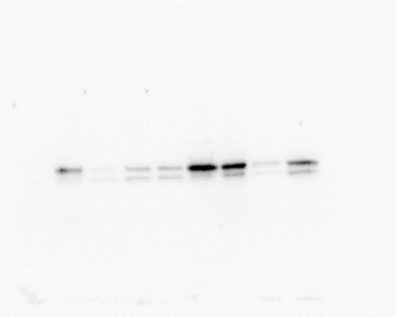

HO-1

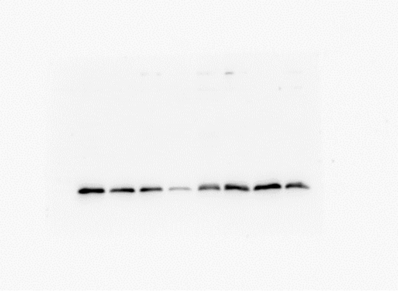

Mfrn-1

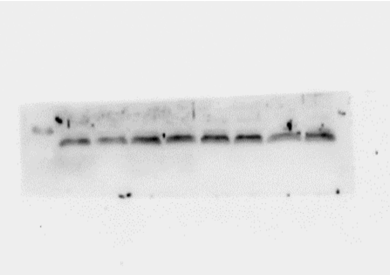

Mfrn-2

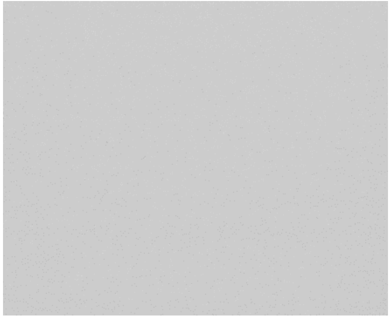

Negative Ctrl DMT-1

Negative Ctrl TfR1

Negative Ctrl FP

Negative Ctrl FECH

Negative Ctrl Mfrn-1

Negative Ctrl Mfrn-2

Negative Ctrl HO-1

Negative Ctrl FTH

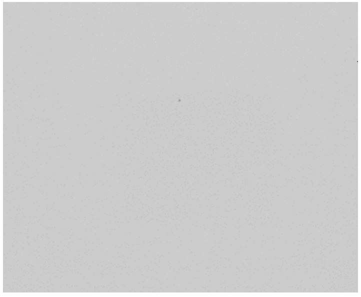

Negative Ctrl GAPDH

Supplementary Figure S1. Original, full-length Western blot images and negative controls (only secondary antibody). The images were taken with a UVItec Alliance Q9 Advanced imaging system using WesternBright ECL chemiluminescent substrate. The negative Ctrls were exposed to light for the same time as in the case of the original WBs.

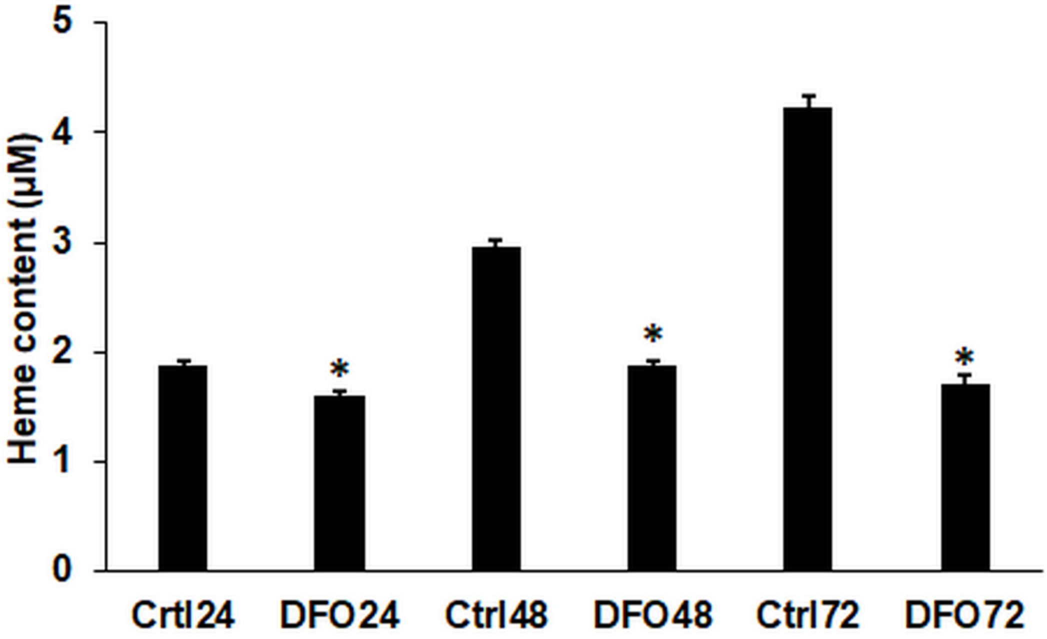

Supplementary Figure S2. Heme concentration measurement in DFO-treated HEC-1A cells. The treatments were carried out in a serum-free environment for 24 h, 48 h, and 72 h. Heme concentration measurements were carried out by a Heme Assay Kit. The measurements were carried out in triplicate in three independent experiments. The columns represent the mean ± SD of three independent experiments (n=3). The asterisk shows $p<0.05$ compared to the control. Abbreviation: DFO-desferrioxamine.
